# Wogonin induces cell cycle arrest and erythroid differentiation in imatinib-resistant K562 cells and primary CML cells

**DOI:** 10.18632/oncotarget.2340

**Published:** 2014-08-10

**Authors:** Hao Yang, Hui Hui, Qian Wang, Hui Li, Kai Zhao, Yuxin Zhou, Yu Zhu, Xiaotang Wang, Qidong You, Qinglong Guo, Na Lu

**Affiliations:** ^1^ State Key Laboratory of Natural Medicines, Jiangsu Key Laboratory of Carcinogenesis and Intervention, Key Laboratory of Drug Quality Control and Pharmacovigilance, Ministry of Education, China Pharmaceutical University, 24 Tongjiaxiang, Nanjing, People's Republic of China; ^2^ Department of Chemistry and Biochemistry, Florida International University, Miami, FL, USA; ^3^ Department of Hematology, The First Affiliated Hospital of Nanjing Medical University; Department of Hematology, The First Affiliated Hospital of Nanjing Medical University, Jiangsu Province Hospital, Nanjing, Jiangsu Province, People's Republic of China

**Keywords:** wogonin, CML, GATA-1, differentiation, cell cycle

## Abstract

Wogonin, a flavonoid derived from *Scutellaria baicalensis* Georgi, has been demonstrated to be highly effective in treating hematologic malignancies. In this study, we investigated the anticancer effects of wogonin on K562 cells, K562 imatinib-resistant cells, and primary patient-derived CML cells. Wogonin up-regulated transcription factor GATA-1 and enhanced binding between GATA-1 and FOG-1, thereby increasing expression of erythroid-differentiation genes. Wogonin also up-regulated the expression of p21 and induced cell cycle arrest. Studies employing benzidine staining and analyses of cell surface markers glycophorin A (GPA) and CD71 indicated that wogonin promoted differentiation of K562, imatinib-resistant K562, and primary patient-derived CML cells. Wogonin also enhanced binding between GATA-1 and MEK, resulting in inhibition of the growth of CML cells. Additionally, in vivo studies showed that wogonin decreased the number of CML cells and prolonged survival of NOD/SCID mice injected with K562 and imatinib-resistant K562 cells. These data suggested that wogonin induces cycle arrest and erythroid differentiation in vitro and inhibits proliferation *in vivo*.

## INTRODUCTION

The K562 cells were derived from a patient suffering from chronic myelogenous leukemia (CML) blast crisis phase and used as the cell line for the research of erythroid differentiation[1, 2]. Erythroid differentiation is a process of formation of red blood cells in the bone marrow is a highly regulated event. The erythrocytes are formed from progenitor cells which is potent to differentiate into erythroid or megakaryocyte lineages. CML is a comprehensive investigation disease. It is a paradigm for neoplasias that evolve through a muti-step process. [3] Patients enter an chronic phase (CP) at first, and then enter an accelerated phase (AP), followed by a blast crisis (BC) during which hematopoietic differentiation becomes arrested[4]. BCR-ABL is a fusion oncogene which encodes tyrosine kinase that is important for the activation of the proliferation and survival pathway[4, 5]. BCR-ABL has been verified to be the causative agent of CML. It is generated by the t(9;22)(q34;q11). Imatinib is a BCR-ABL kinase inhibitor which blocks the growth of BCR-ABL-transformed cells, and is effective in disease remission in chronic phase CML patients [6-8]. However, resistance to imatinib develops that ultimately leads to disease progression[9]. Recently, results from the ‘non-randomized stop IM’ trial showed that 61% of CML patients who discontinued imatinib after achieving a complete molecular remission eventually relapsed [10-12]. Therefore, a great deal of attention has been paid to develop alternative molecular-based strategies for treatment of imatinib-resistant CML.

GATA-1 is a zinc finger transcription factor that induces the differentiation of megakaryocytes and erythrocytes, and is required for mast cell and eosinophil development[13, 14]. Recent studies have found that GATA-1 directly represses transcription of numerous genes (including GATA-2 and c-Kit), and inhibits cell division during terminal hematopoietic differentiation [15, 16]. It is reported that GATA-1 plays critical roles for granulocyte/monocyte (GM) and megakaryocyte/erythrocyte (MegE) lineage commitment. GATA-1 is critical for the development of megakaryocytes and erythroid cells[17-19]. In addition to GATA-1, Friend of GATA-1 (FOG-1), is also required for differentiation of megakaryocytes and erythrocytes, where GATA-1 is expressed, and collaborates with GATA-1 to induce erythroid differentiation [14, 17, 20]. Regulation of GATA-1 chromatin occupancy is critical for its control of hematopoietic lineage specification by FOG-1[14].

Wogonin is a natural flavonoid derived from a Chinese herb, *Scutellaria baicalensis*, and has been reported to induce differentiation of AML cells [21, 22]. The ability to induce cell differentiation may be an important attribute for an anti-leukemia agent, and we therefore further explored the effects of wogonin on induction of differentiation in CML cells. We investigated the effect of inducing differentiation, cycle arrest and growth inhibition of wogonin on K562, K562 imatinib-resistant cells (K562r), and primary patient-derived CML cells in vitro. Also we detected the anti-CML effect of wogonin *in vivo*. To further investigate the mechanism of wogonin in anti-CML effect, we studied the effect of wogonin on GATA-1. Our results about GATA-1 were consistent with those in previous reports, and it was possible that wogonin induced differentiation, arrested cell cycle and inhibited growth of CML cells by regulating GATA-1. Our data suggest that wogonin may be useful for clinical treatment of CML patients.

## RESULTS

### Effects of wogonin on K562 and K562r cell cycle and differentiation

**Figure 1:**
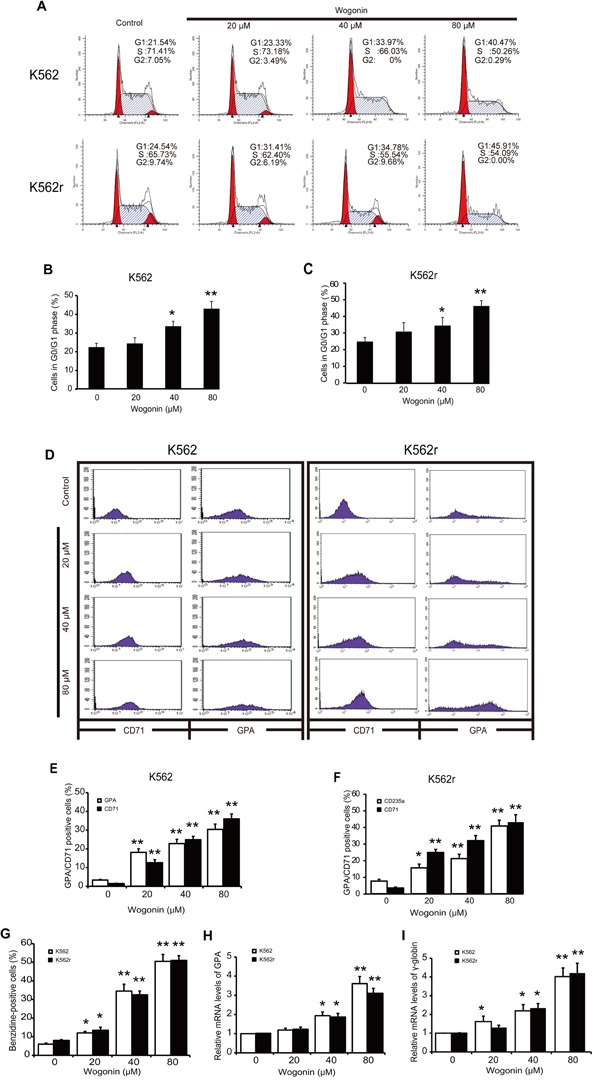
Cell cycle arrest induction and differentiation induction effects of wogonin on K562 and K562r cells (A) Cell cycle analysis of K562 and K562r cells treated with 20, 40, and 80 μM wogonin for 48 hours was performed by flow cytometry. (B, C) The percentages of G0/G1 phase cells following wogonin treatment for 48 hours are shown. Data represent mean ± SEM from 3 independent experiments. (D) The percentages of cells expressing CD71 and GPA were detected by flow cytometry analyses. (E, F) The data represent the mean ± SEM of 3 different experiments. Asterisks denote statistically significant (P <0.05) differences compared with controls by one-way ANOVA. (G) Benzidine-positive cells with brown-blue color were counted. The percentage was calculated based on 200 total cells per microscopic field and counting 5 times in each group. (H, I) GPA and γ-globin mRNA levels were detected by quantitative real-time reverse transcription-PCR and fold changes were normalized to β-actin. Asterisks denote significant (P <0 .05) differences compared to controls by two-tailed Student's t test

According the pre-experiment, we applied wogonin in the concentrations of 20 μM, 40 μM, and 80 μM. Results showed that wogonin arrested cell cycle at the G0/G1 phase in K562 cells (Figure 1A-B). A 4-day incubation with wogonin exerted a dose-dependent effect on differentiation of K562 cells as judged by benzidine staining (Figure 1G). To further confirm the effect of wogonin on K562 cell differentiation, we examined the expression of GPA and CD71, two erythroid differentiation specific markers. As shown in Figure 1D and 1E, results showed that the numbers of GPA and CD71 positive cells increased following incubation with wogonin for 96 hours. And the mRNA level of GPA increased (Figure 1H). Additionally, the mRNA level of γ-globin increased, one erythroid differentiation relative gene, in the cells following incubation of wogonin for 96 hours. The dose-dependent increase of γ-globin further demonstrated the differentiation inducing activity of wogonin in K562 cells (Figure 1I). These results indicated that wogonin induced differentiation and arresting cell cycle at G0/G1 phase in K562 cells.

Interestingly, we found the similar effect in imatinib-resistant K562 (K562r) cells. Imatinib mesylate (Gleevec, STI571), a first generation oral BCR-ABL kinase inhibitor, blocks the growth of BCR-ABL -transformed cells, and is highly effective in inducing disease remission in chronic phase CML patients (remission rate > 90%) [23, 24]. Unfortunately, the pre-existence of imatinib mesylate resistance ultimately leads to therapy failure[6, 8]. Wogonin exerted its anticancer effects via a different mechanism from imatinib, suggesting the possible sensitivity of K562r cells. Wogonin arrested cell cycle at G0/G1 phase in K562r cells (Figure 1A, 1C). Benzidine staining, specific markers detection of GPA and CD71 and mRNA expression of GPA and γ-globin results showed the differentiation inducing activity of wogonin (Figure 1D, 1F-I). These results showed that wogonin induced erythroid differentiation and arrest cell cycle in both K562 and K562r cells.

### Effects of wogonin on GATA-1 regulation in K562 and K562r cells

**Figure 2:**
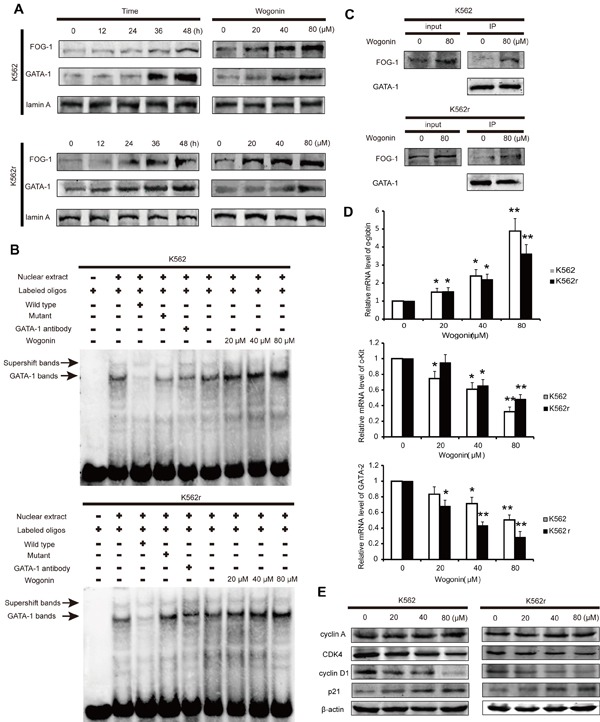
Wogonin regulates the expression of GATA-1 and p21 in K562 and K562r cells (A) Expression levels of GATA-1 and FOG-1 were analyzed by western blotting. Lamin A was used as a loading control. (B) EMSA assay detecting GATA-1 binding to its consensus site. Cells were incubated with wogonin for 48 hours and DNA binding was determined in nuclear extracts using EMSA. To determine the composition of the DNA-binding complex, the anti-GATA-1 antibody was used for supershift experiments. Data are representative of 3 separate experiments. (C) Cells incubated with/without 80 μM wogonin for 36 hours were immunoprecipitated with anti-GATA-1 antibody, followed by western blot analysis with anti-FOG-1 antibodies. (D) α-globin, c-kit and GATA-2 mRNA levels were detected by quantitative real-time PCR, and fold changes were assessed and shown normalized to β-actin. Asterisks denote significant (P <0 .05) differences relative to controls by two-tailed Student's t test. (E) Expression levels of cyclin A, CDK4, cyclin D1 and p21 were analyzed by western blotting with β-actin as a loading control. Data are representative of 3 independent experiments.

To further examine the mechenism of wogonin on K562 and K562r cells, we detected nuclear proteins expression in these two cell lines in the presence of wogonin for 0, 12, 24, 36, and 48 hours. Western blot analyses showed that levels of nuclear GATA-1 and FOG-1 were increased in a dose-dependent manner following incubation with wogonin (Figure 2A). Moreover, cells treated with 20, 40, and 80 μM wogonin for 48 hours showed a dose-dependent increase in DNA binding of GATA-1 (Figure 2B). It has been reported that FOG-1 and GATA-1 are coexpressed during embryonic and hematopoietic development[25, 26]. GATA-1 binds to FOG-1 and synergistically activates transcription from a hematopoietic-specific regulatory region[25-28]. We therefore detected the binding ability between GATA-1 and FOG-1 following incubating with wogonin for 48 hours (Figure 2C). The immunoprecipitation (IP) assay showed that wogonin enhanced the binding ability between GATA-1 and FOG-1, which was confirmed by real-time PCR analysis of α-globin, c-kit and GATA-2, the genes regulated by GATA-1 (Figure 2D). Therefore, it is concluded that wogonin induced erythroid differentiation by increasing expression of GATA-1 and FOG-1 and modulating DNA transcription via up-regulation of the binding of FOG-1 to GATA-1.

### Effect of GATA-1 on cell cycle in K562 and K562r cells

It is reported that GATA-1 binds to the promoter of p21 and activates its transcription[29-32]. To investigate the molecular mechanism of cell cycle arrest induced by wogonin, we examined the levels of the key checkpoints of cell cycle progression in K562 and K562r cells. The ChIP experiment showed that the binding ability between GATA-1 and the promoter of p21 in both K562 and K562r cells enhanced when treated wogonin for 48 hours (Supplement figure 1). Also, cells treated with wogonin showed up-regulation of p21 (Figure 2E). Furthermore, wogonin treatment down-regulated cyclin-dependent kinase 4 (CDK4) and cyclin D1 (Figure 2E). These results showed that wogonin arrested cell cycle at G0/G1 phase by regulating GATA-1 associated cell cycle checkpoints in K562 cells.

### Antiproliferative effects of wogonin *in vivo*

**Figure 3:**
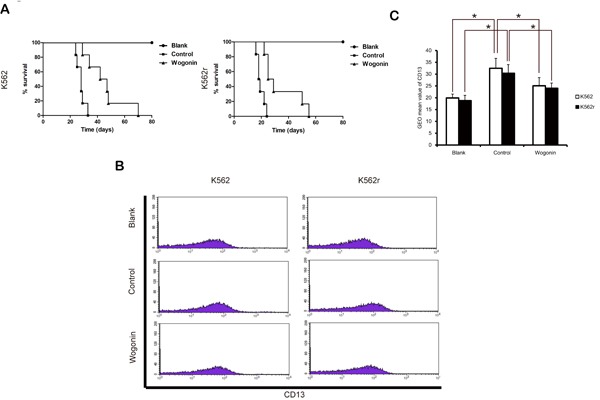
Effects of wogonin on CML-bearing mice (A) A Kaplan-Meier survival plots of CML-bearing NOD/SCID mice are shown. K562 and K562r cells were injected through tail vein 24 hours (2-5×10^6^ cells per mouse, 6 mice per group) after sublethally irradiation (2.4 Gy). Data are representative of 2 separate experiments. Animals were observed for 60 days after cell injection. The survival curves differed significantly between the wogonin-treated group and the control group. Wogonin prolonged survival in mice compared with controls (P<0.001; log-rank test). (B) CD13 expression was examined in blood samples from three mice of each group. (C) Data represent the mean ± SEM of 3 different experiments. Asterisks denote statistically significant (P<0.05) differences compared with controls by one-way ANOVA.

We next examined the effects of wogonin *in vivo*. 15 days after injection with K562 and K562r cells, blood samples from three mice randomly in each group were examined for CD13 expression, a surface marker of K562 cells. The results showed that mice inoculated K562 and K562r cells expressed more CD13 than normal mice and wogonin significantly decreased the expression of CD13 in K562 and K562r group (Figure 3B-C). Wogonin also extended the survival of CML-bearing mice compared with mice in the control group (Figure 3A). The median survival time of mice injected with K562 and K562r cells were 27.8±2.91 and 19.5±2.7 days in the control groups and were 45±13.1 and 34.3±13.1 days in the wogonin-treated groups. These results demonstrated that wogonin suppresses proliferation of K562 and K562r cells *in vivo*.

### Effects of wogonin on differentiation of primary CML cells

**Figure 4:**
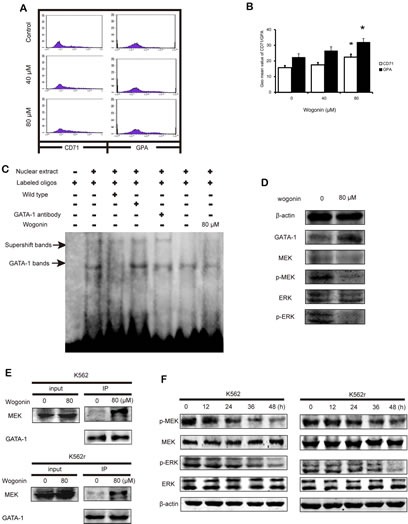
Wogonin induces differentiation on primary CML cells and increases the interaction between GATA-1 and MEK (A) The expression of CD71 and GPA was detected by flow cytometry analyses. Primary CML cells were treated with wogonin (40 and 80 μM) for 48 hours. (B) Quantification of the data shown in A. Data represented the mean ± SEM of 3 different experiments. Asterisks denote statistically significant (P <0.05) differences compared with controls by one-way ANOVA. (C) EMSA assay detected GATA-1 binding to its consensus site. Primary CML cells were incubated with wogonin for 48 hours, and DNA binding was determined in nuclear extracts using EMSA. To determine the composition of the DNA-binding complex, the anti-GATA-1 antibody was used for supershift experiments. Data are representative of 3 independent experiments. (D) Expression levels of GATA-1, MEK, p-MEK, ERK and p-ERK were analyzed by western blotting using indicated antibodies. β-actin was used as a loading control in primary CML cells. (E) Cells incubated with/without 80 μM wogonin for 36 hours were immunoprecipitated with anti-GATA-1 antibody, followed by western blot analysis with anti-MEK antibodies. (F) Expression levels of MEK, p-MEK, ERK and p-ERK were analyzed by western blotting using indicated antibodies. β-actin was used as a loading control.

Primary leukemic cells from CML patients were collected and used to investigate the effects of wogonin in CML cells. As shown in Figure 4A and 4B, expression of CD71 and GPA in CML cells increased following treatment with wogonin for 96 hours (Figure 4A-B). These data showed that wogonin induced differentiation in primary CML cells. The western blot results showed that the expression of GATA-1 increased in wogonin-treated primary CML cells (Figure 4D). However, results of electrophoretic mobility shift assay (EMSA) showed no obvious difference between the control group and wogonin-treated group in the ability of DNA binding (Figure 4C). These results showed that GATA-1 has other effects on primary CML cells.

### Wogonin regulates the effect of GATA-1 and inhibits phosphorylation of MEK and ERK in K562, K562r, and primary CML cells

We next investigated the mechanism by which wogonin inhibits proliferation of K562, K562r, and primary CML cells. We speculated that GATA-1 may inhibit cell proliferation through a non-transactivation mechanism. It is known that continued activation of the Philadelphia chromosome causes phosphorylation of downstream proteins such as MEK and ERK, resulting in activation of cell proliferation pathways[33, 34]. In addition, it has been reported that GATA-1 inhibits the MEK pathway by binding with MEK[35]. Therefore, we speculated that wogonin affects the binding ability between GATA-1 and MEK. Following treatment with wogonin for 36 hours, IP results showed that wogonin enhanced the binding ability between GATA-1 and MEK in K562 and K562r cells (Figure 4E), and then we examined the phosphorylation of MEK and ERK in these two cell lines. The results showed that levels of phosphorylated MEK and ERK decreased following incubation with wogonin (Figure 4F). The primary CML cells treated with wogonin for 48 hours also showed notable reduction of phosphorylated MEK and ERK (Figure 4D). These results suggested that, apart from increasing expression of p21 and promoting DNA binding, wogonin increased protein expression of MEK and ERK by enhancing the binding ability between GATA-1 and MEK.

### Role of GATA-1 in wogonin-induced differentiation and cycle arrest effect on K562 and K562r cells

**Figure 5:**
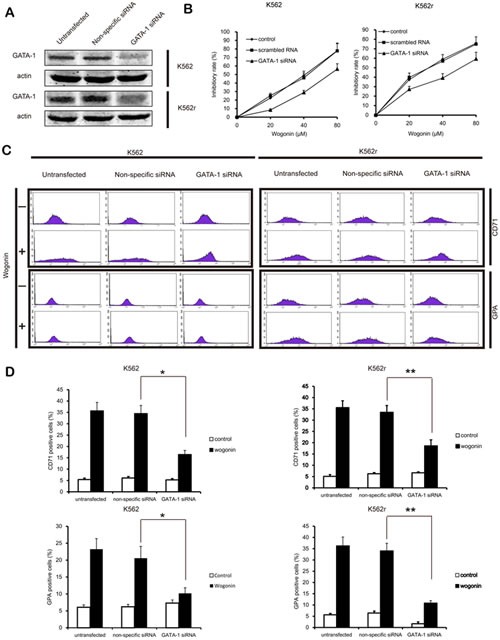
GATA-1 is involved in wogonin-modulated growth inhibition and differentiation (A) Following siRNA transfection, K562 and K562r cells were treated with or without wogonin for 96 hours. Confirmation of the silencing of GATA-1 expression was detected by western blotting with β-actin as a loading control. (B) Growth inhibition of wogonin was assessed by MTT assay. (C) CD71 and GPA-positive cells were measured by flow cytometry. (D) The data represent the mean ± SEM of 3 different experiments. Asterisks denote statistically significant (P <0.05) differences compared with controls by one-way ANOVA.

**Figure 6:**
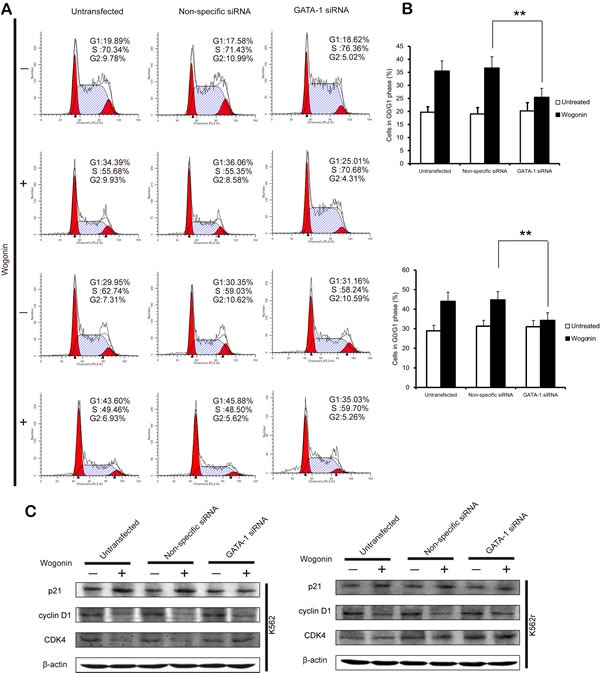
GATA-1 is involved in wogonin-modulated cycle arrest (A) The percentages of cells in G0/G1 phases of the cell cycle are shown. (B) Data represent the mean ± SEM of 3 different experiments. Asterisks denote statistically significant (P <0.05) differences compared with controls by one-way ANOVA. (C) Effect of silencing GATA-1 on the expression of cell cycle checkpoint proteins.

To better understand the role of GATA-1 in wogonin-induced differentiation and cell cycle arrest, we transfected the siRNA of GATA-1 into K562 and K562r cells and monitored efficacy of transfection using western blot assays. Results of MTT assay showed that GATA-1 siRNA partially reduced wogonin-induced cell proliferation inhibition (Figure 5B). After silencing GATA-1 (Figure 5A), wogonin-induced expression of CD71 and GPA in K562 and K562r cells decreased (Figure 5C-D). In the same way, with the GATA-1 silence, the effect of wogonin on cell cycle arrest was also inhibited (Figure 6A-B). Meanwhile, the regulative effect of wogonin on the levels of cell cycle regulators were also reduced (Figure 6C). These data suggested that GATA-1 is required for wogonin-induced cell differentiation and cell cycle arrest.

## DISCUSSION

Treatment of leukemia by forcing malignant cells to undergo differentiation instead of being killed by cytotoxic drugs is a promising alternative therapeutic strategy for leukemia, which has been previously applied for treatment of AML[36, 37]. In this study, we found an interesting phenomenon that wogonin could induce erythroid differentiation in K562 cells and primary CML cells, demonstrated by the benzidine staining assay and increased level of CD71 and GPA.

GATA-1 is a transcription factor that regulates cellular differentiation and proliferation[38, 39]. It is initially discovered as a nuclear protein that binds numerous GATA consensus motifs distributed throughout various enhancers and promoters of erythroid-specific genes[38]. Moreover, GATA-1 is also a key regulator of erythropoiesis, and inhibition of GATA-1 decrease the erythropoietic effect of EPO[40]. The essential GATA-1 cofactor FOG-1 is required for differentiation of erythrocytes, and recent studies have shown that FOG-1 promotes GATA-1 transcription activity not only by recruiting coactivators, but also by directly modulating chromatin occupancy[38, 41]. Our results demonstrated that wogonin enhances DNA binding of GATA-1 and recruits FOG-1, resulting in increased expression of erythroid-specific genes, leading to erythroid differentiation. The results of siRNA confirmed that GATA-1 has an important role in wogonin-induced erythroid differentiation. GATA-1 is the key factor involved in wogonin-induced differentiation. There are many factors that affect the expression of GATA-1 including the GATA-1/GATA-2 switch effect[41]. Other signaling pathways, such as the PI3K/AKT pathway, may regulate the phosphorylation and subsequent activity of GATA-1. It is previously reported that wogonin has the ability to activate the PI3K/AKT pathway[42]. Further studies are required to study whether wogonin regulates erythroid differentiation through other signaling pathways.

Cell cycle arrest is an essential early event in inducing differentiation. Therefore, we detected the cell cycle arrest effect of wogonin on K562 cells. Also, we focused on the effect of GATA-1 in cell cycle arrest. Previous research showed that GATA-1 binds to the promoter of p21 and activates the expression of p21 and its downstream proteins[29]. Our data showed that wogonin arrested the cell cycle following 48 hours treatment by enhancing the binding ability between GATA-1 and the promoter of p21 and then promoted the expression of p21.

We had demonstrated that wogonin induced erythroid differentiation and cycle arrest and found that wogonin had these effects via regulating the function of GATA-1. *In vivo* results showed that wogonin prolonged the survival of mice injected with CML cells by inhibiting proliferation of K562 cells. Our investigation indicated that wogonin inhibited proliferation of K562 cells via GATA-1. Wogonin increased the binding ability of MEK and GATA-1, inhibiting the activation of the MEK-ERK pathway[35]. GATA-1 inhibits phosphorylation of ERK by interacting with MEK, which is upstream of ERK, and the phosphorylation of ERK inhibits cell proliferation[35]. It has been reported that MEK contains a nuclear export signal in its N-terminal domain, indicating that MEK works in the nucleus for signaling purposes, and then returns to the cytoplasm. GATA-1 binds with MEK in the nucleus and inhibits its activity[43]. Wogonin increased the binding ability between GATA-1 and MEK, and then inhibited phosphorylation of MEK. Therefore, we knew that, GATA-1 is the key factor in wogonin's effect on K562 cells.

We further examined the effect of GATA-1 on primary CML cells. Our studies showed that levels of phosphorylated MEK and ERK in primary CML cells were higher than those in K562 cells, and wogonin showed a more inhibitory effect on phosphorylation of MEK and ERK in primary CML cells (Data not shown). This difference might be due to the fact that the primary cells used in the current study were blast crisis cells. Although K562 is a blast crisis cell line, the primary cells may show a stronger ability in proliferation. Therefore, primary cells have aberrant copy numbers of BCR-ABL, which is likely to provide stronger survival signaling[10]. Finally, wogonin did not affect DNA binding in primary CML cells, which was totally different from the activity in K562 cells, suggesting that GATA-1 plays a different role under different cellular environments.

Depart from K562 cells we also focused on the effect of wogonin on K562r cells. Drug-resistance presents a significant problem when using imatinib for the treatment of CML patients. The resistance of CML to imatinib treatment mostly manifests as decreased drug uptake and mutation of the BCR-ABL fusion gene[5]. Although the second generation TKIs, such as nilotinib and dasatinib, appear to be effective in imatinib-resistant patients, these drugs are also TKIs, it is possible for these drugs appear the similar resistance with imatinib, and therefore new treatment strategies are needed urgently[5, 9, 44]. Recently, results from the ‘non-randomized stop IM’ trial showed that 61% of CML patients who discontinued imatinib after achieving a complete molecular remission eventually relapsed[10, 11]. For example, the Hsp90 inhibitor geldanamycin selectively sensitizes Bcr-Abl-expressing leukemia cells[12]. The mechanism by which wogonin inhibits proliferation of CML cells is totally different from that of imatinib, which offers a possibility that wogonin may be effective in imatinib-resistant CML. Our *in vivo* and *in vitro* data showed that wogonin induced differentiation and cell cycle arrest in K562r cells, inhibited cell proliferation, and extended lifespan of K562r-bearing mice. These findings strongly suggested that wogonin may be an alternative drug for treatment avoiding the drug-resistance problem associated with TKIs.

In conclusion, our study showed that wogonin induced erythroid differentiation and cell cycle arrest in CML cells via regulating the function of GATA-1. Wogonin increased the expression of GATA-1 then activated transcription and promoted the expression of p21 and enhanced the binding ability between GATA-1 and MEK. Additionally, wogonin significantly prolonged survival of CML-bearing mice by inhibiting proliferation of K562 and K562r cells. These data suggested that wogonin is a potent drug for treatment of CML. Moreover, because its mechanisms of action differ from those of TKIs, wogonin may provide an alternative for TKI-resistance CML. In this study, we found that the dosage is high both *in vivo* and *in vitro*, we think that wogonin may become an assist drug for CML and combine with TKIs.

## MATERIALS AND METHODS

### Reagents and antibodies

Wogonin (purity ≥99%) was synthesized and used in all experiments unless otherwise indicated[45]. The final concentration of dimethyl sulfoxide (DMSO) in all experiments was ≤0.01%, and cells treated with the highest concentration of DMSO were used as controls in the corresponding experiments. Heat-inactivated fetal bovine serum (FBS), and RPMI 1640 medium were purchased from Gibco Invitrogen Corporation (Carlsbad, CA, USA). PE anti-human CD71 (Transferrin Receptor), APC anti-human Glycophorin A (CD235a) and FITC anti-human CD13 antibodies were purchased from eBioscience (San Diego, CA, USA). Antibodies against GATA-1, FOG-1, MEK, ERK, p-ERK (Thr 177/160), and β-actin were purchased from Santa Cruz Biotechnology (Santa Cruz, CA, USA), and antibodies against p-MEK (Ser 217/221) were purchased from Cell Signaling Technology (Danvers, MA, USA). IRDyeTM 800-conjugated secondary antibodies were obtained from Rockland (Philadelphia, PA, USA).

### Cell culture

The K562 cell line was purchased from the cell bank of Shanghai Institute of Biochemistry and Cell Biology. Imatinib-resistant K562 cells were kindly provided by Professor Junia V. Melo, Department of Pathophysiology, Chemical Biology Division of Shanghai Universities E-Institutes, Key Laboratory of Cell Differentiation and Apoptosis of the National Ministry of Education. Primary leukemic cells from CML blast crisis patients (Zhongda Hospital of Southeast University, Nanjing, China) were collected using lymphocyte-monocyte separation medium (Jingmei, Nanjing, China). The protocol of collection of cells from patients complied with guidelines in the Declaration of Helsinki, and was approved by the Institutional Review Board of Zhongda Hospital. A signed informed consent was obtained from each patient. Primary leukemic cells and K562/K562r cell lines were cultured with RPMI 1640 medium supplemented with 10% FBS.

### Cell cycle analysis

The cell cycle analysis was employed by propidium iodide staining and quantified by a FACS Calibur flow cytometer (Becton, Dickinson, San Jose, CA, USA)[46]. The percentage of cells in each phase of the cell cycle was analyzed with Modfit software (Becton, Dickinson, San Jose, CA, USA).

### Animal model

Animal studies were conducted in accordance with the regulations of the China Food and Drug Administration (CFDA) on Animal Care. Female NOD/SCID immunodeficient mice (aged 6–9 weeks) were purchased from Beijing HFK bioscience Company Limited. The mice were maintained in an air-conditioned pathogen-free room under conditions of controlled lighting (12 h light/day) and fed a standard diet of laboratory food and water. These immunodeficient mice were sublethally irradiated (2.4 Gy), and were injected with K562, K562r, or primary CML cells via the tail vein (2–5 × 10^6^ cells per mouse, n = 6 per group) in 24 hours following the radiation treatment. Animals in the control group were injected with Physiological saline to evaluate the effects of injection on survival. The next day, the mice were injected intraperitoneally (i.p.) with or without wogonin (80 mg/kg) every other day for 14 days[42]. Following i.p. injection, the animals in K562 and K562r groups were inspected daily for 80 days, the animals in primary CML group were inspected daily for 30 days, and survival was represented using a Kaplan-Meier survival plot.

### Cell proliferation assays *in vivo*

*In vivo* investigations were performed using immunodeficient (NOD/SCID) mice engrafted with, K562, K562r, or primary human CML cells. Twenty days later, blood of the NOD/SCID mice was collected and the expression of CD13, a marker of K562, was examined by flow cytometry with a FACS Calibur flow cytometer (Becton, Dickinson, San Jose, CA, USA).

### Cell differentiation analysis

Cells (5 × 10^4^ cells/mL) were incubated with different concentrations of wogonin in 6-well flat bottom plates for 5 days. Following incubation, the cells were collected and washed twice with PBS, stained with benzidine solution containing 3% H_2_O_2_ for 30 min, followed by addition 0.6 mL of 10% ethanoic acid[47]. Cells displaying brown-blue color after staining were regarded as erythroid differentiation positive cells. The experiment was conducted in triplicate. Expression of the cell surface differentiation markers GPA and CD71 was determined by flow cytometry with a FACS Calibur flow cytometer (Becton, Dickinson, San Jose, CA, USA).

### Western blot analysis

The Western blot analysis was prepared as described previously[48]. Cells were collected and lysed in lysis buffer (50 mM Tris-Cl, pH 7.6, 150 mM NaCl, 1 mM EDTA, 1% (m/v) NP-40, 0.2 mM phenylmethanesulfonyl fluoride (PMSF), 0.1 mM NaF, and 1.0 mM dithiothreitol). The lysates were centrifuged at 13,000 × g and 4 °C for 15 min. Protein concentrations in the supernatants were measured using a bicinchoninic acid (BCA) assay kit (Pierce, Rockford, IL, USA) by a Varioskan multimode microplate spectrophotometer (Thermo Waltham, MA, USA). Equal amounts of protein (50 μg) were separated by 8-12% sodium dodecyl sulfate polyacrylamide gel electrophoresis (SDS-PAGE) and transferred onto PVDF membranes (Millipore, Boston, MA, USA). The blots were then incubated with primary antibodies for overnight at 4°C, followed by incubation with IRDyeTM800 conjugated secondary antibody for 1 h at 37°C. Detection was performed using the Odyssey Infrared Imaging System (LI-COR Inc, Lincoln, NE, USA).

### Immunoprecipitation

Supernatants of cell lysates were incubated with GATA-1 antibody for 1 h at 4 °C, and then added 20 μl of protein G/A agarose beads (Santa Cruz Biotechnology, St. Louis Park, Minnesota, US) overnight at 4 °C. Beads were washed with cell lysis buffer 4 times and bound proteins were eluted with 2× loading sample buffer and analyzed by Western blot with indicated antibodies.

### Electrophoretic mobility shift assay (EMSA)

Nuclear extracts were prepared and electrophoretic mobility shift assay was conducted according to the manufacturer's instructions (EMSA kit; Beyotime, Nanjing, China)[49].

### Quantitative real-time reverse transcription-polymerase chain reaction (RT-PCR)

The RT-PCR assay was prepared as described previously[48]. For quantitative analysis of gene expression, total RNA was isolated using a TRIzol kit (Invitrogen, Carlsbad, CA, USA). Complementary DNA was synthesized using a complementary DNA synthesis kit (Code DRR047A; TaKaRa, Kyoto, Japan) according to the manufacturer's instructions. Relative quantities of mRNA were measured using Applied Biosystems 7500 Fast Real-Time PCR System (PerkinElmer, Torrance, CA, USA) and double-stranded DNA dye SYBR Green PCR core reagents (Code DRR081C; TaKaRa, Kyoto, Japan). Amplification was performed with 40 cycles at 95°C for 15 seconds and then at 60°C for 30 seconds. Data were analyzed using 7500 system SDS software.

### Statistical analysis

All data are expressed as the mean ± standard error of the mean (SEM) for the values obtained from at least three independent experiments. Statistical analysis of multiple-group comparisons was performed by one-way analysis of variance (ANOVA) followed by the Bonferroni posthoc test. Comparisons between two groups were analyzed using the two-tailed Student's t-test. The survival of NOD/SCID mice was evaluated by Kaplan-Meier analysis using the log-rank test to compare differences. P value <0.05 was considered statistically significant.

## SUPPLEMENTARY MATERIAL AND FIGURES


