# Correction: Wogonin induces cell cycle arrest and erythroid differentiation in imatinib-resistant K562 cells and primary CML cells

**DOI:** 10.18632/oncotarget.27373

**Published:** 2020-01-21

**Authors:** Hao Yang, Hui Hui, Qian Wang, Hui Li, Kai Zhao, Yuxin Zhou, Yu Zhu, Xiaotang Wang, Qidong You, Qinglong Guo, Na Lu

**Affiliations:** ^1^ State Key Laboratory of Natural Medicines, Jiangsu Key Laboratory of Carcinogenesis and Intervention, Key Laboratory of Drug Quality Control and Pharmacovigilance, Ministry of Education, China Pharmaceutical University, 24 Tongjiaxiang, Nanjing, People’s Republic of China; ^2^ Department of Chemistry and Biochemistry, Florida International University, Miami, FL, USA; ^3^ Department of Hematology, The First Affiliated Hospital of Nanjing Medical University, Jiangsu Province Hospital, Nanjing, Jiangsu Province, People’s Republic of China

**This article has been corrected:** Due to errors in figure preparation, there are duplicate pictures of the K562 cells between 0 and 20 μM wogonin treatment groups in Figure 1A. The corrected Figure 1 is shown below. The authors declare that these corrections do not change the results or conclusions of this paper.

Original article: Oncotarget. 2014; 5:8188–8201. 8188-8201. https://doi.org/10.18632/oncotarget.2340

**Figure 1:**
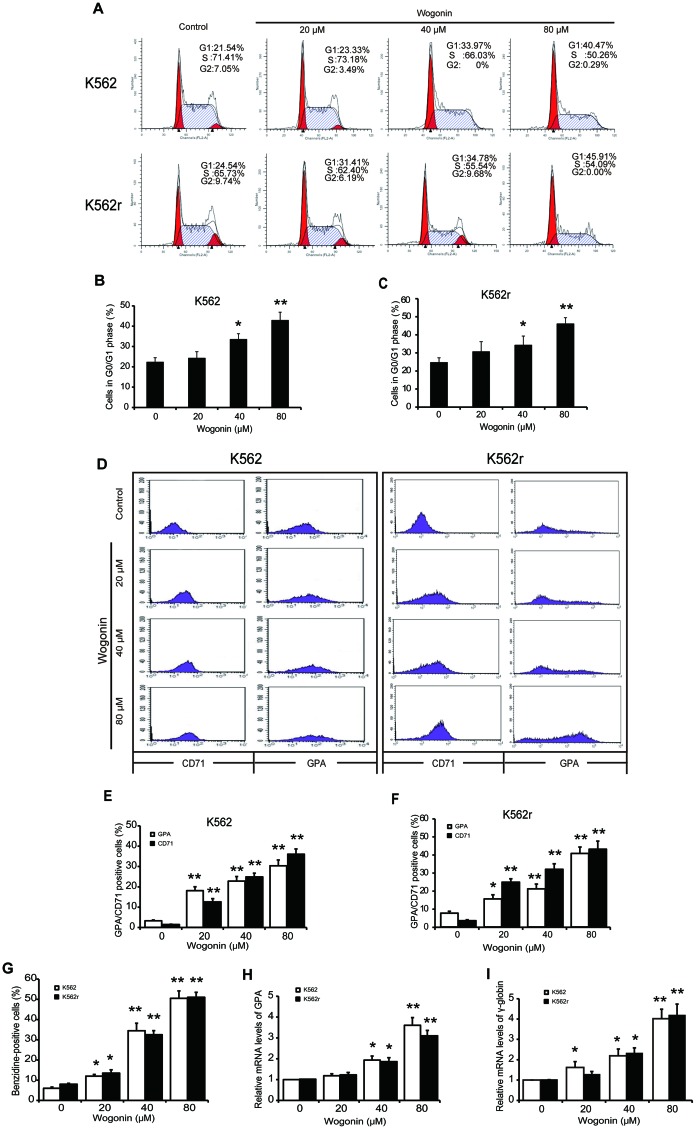
Cell cycle arrest induction and differentiation induction effects of wogonin on K562 and K562r cells.

(A) Cell cycle analysis of K562 and K562r cells treated with 20, 40, and 80 μM wogonin for 48 hours was performed by flow cytometry. (B, C) The percentages of G0/G1 phase cells following wogonin treatment for 48 hours are shown. Data represent mean ± SEM from 3 independent experiments. (D) The percentages of cells expressing CD71 and GPA were detected by flow cytometry analyses. (E, F) The data represent the mean ± SEM of 3 different experiments. Asterisks denote statistically significant (P <0.05) differences compared with controls by one-way ANOVA. (G) Benzidine-positive cells with brown-blue color were counted. The percentage was calculated based on 200 total cells per microscopic field and counting 5 times in each group. (H, I) GPA and γ-globin mRNA levels were detected by quantitative real-time reverse transcription-PCR and fold changes were normalized to β-actin. Asterisks denote significant (P <0 .05) differences compared to controls by two-tailed Student’s t test.

