# Supplementary material for: Wogonin induces cell cycle arrest and erythroid differentiation in imatinib-resistant K562 cells and primary CML cells
Source: Oncotarget. 2014 Aug 10;5(18):8188–201. doi: 10.18632/oncotarget.2340 (PMC4226676; doi:10.18632/oncotarget.2340)
Supplement: Supplementary file 1 [file oncotarget-05-8188-s001.pdf]

Wogonin induces cell cycle arrest and erythroid differentiation in imatinib-resistant K562 cells and primary CML cells

Supplementary Material

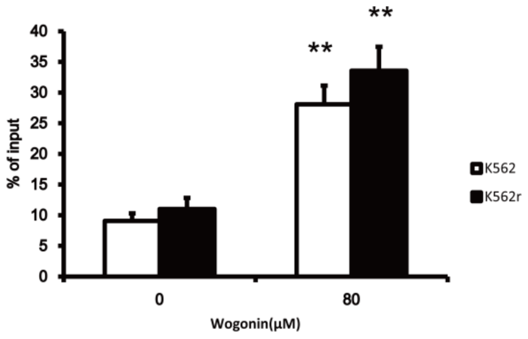

Supplementary Figure 1:

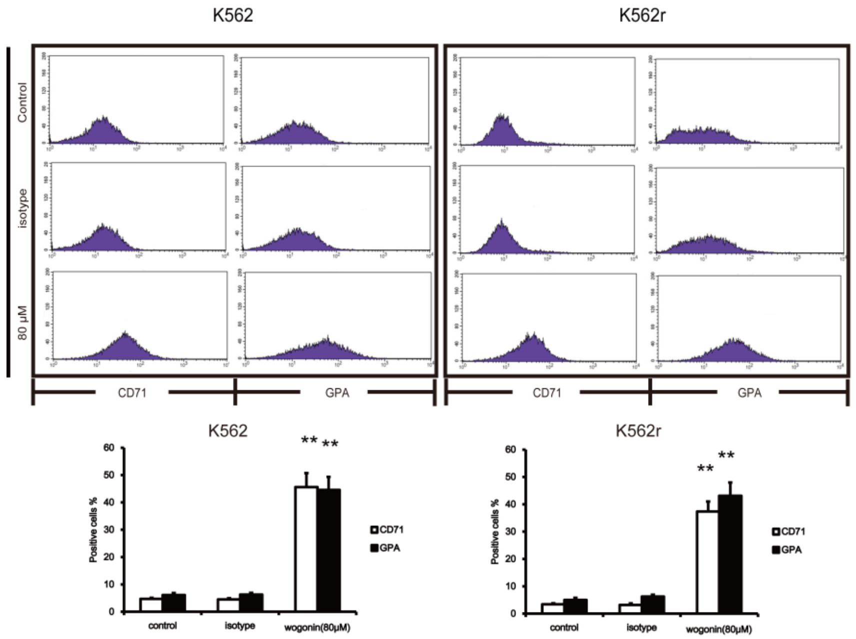

Supplementary Figure 2:

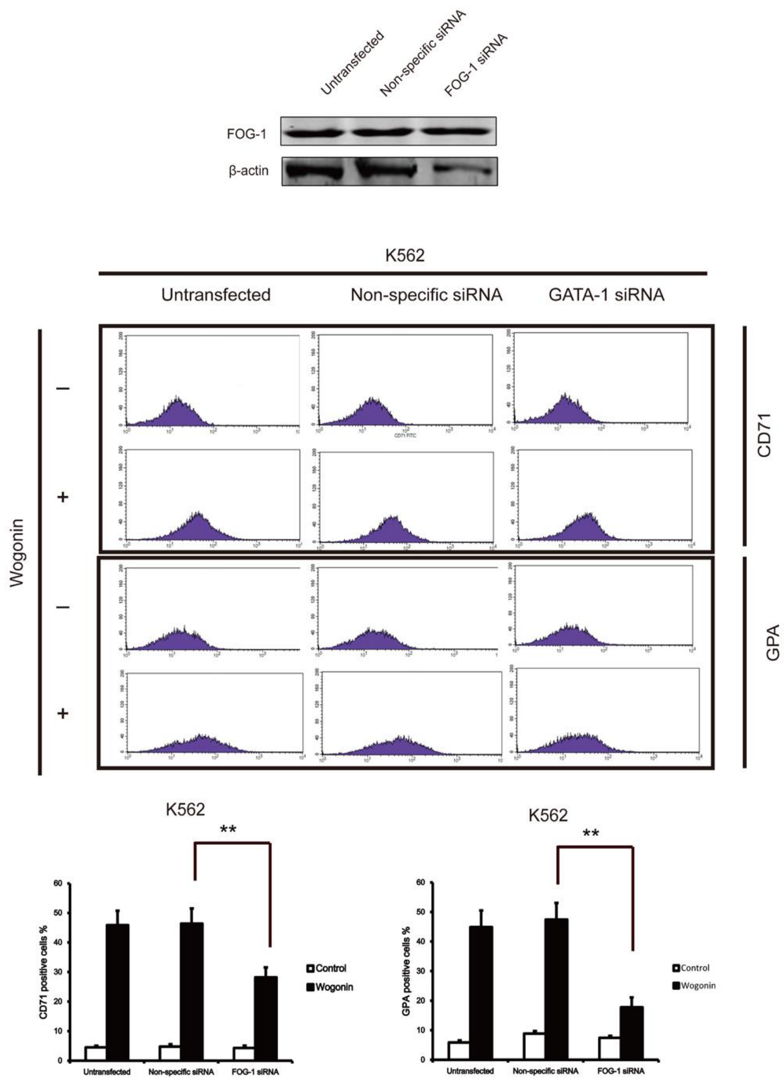

Supplementary Figure 3:

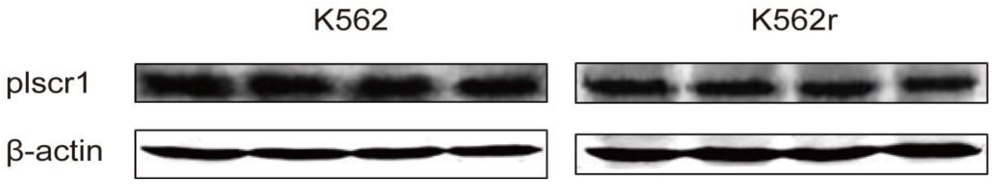

Supplementary Figure 4:
